# Clinical Experiences in Private Practice

**Published:** 1873-04

**Authors:** Z. Collins McElroy

**Affiliations:** Zanesville, Ohio


BUFFALO

### Medical and Surgical Journal.

VOL XII. APRIL, 1873. No. 9

### Original Communications.

:o:

ART. I.—*Clinical experiences in private practice. The Realien* of a case of so called phlegmonous erysipelas, or moist gangrene.* By Z. COLLINS MCELROY, M. D., Zanesville, Ohio.

Reported to Muskingum County Medical Society, at its session in the City of Zanesville, Ohio. February 6th, 1873.

B. W., aged 70, weight 170, height 5 8½; a well preserved, and well provided for man in every respect. Has been for many years a leading business man in our city; known to all of us for his rather extraordinary activity, as he could be seen almost any day on our streets; sometimes in a saddle, sometimes in a carriage, but more frequently on foot, and that without much regard to the weather. Always absorbed in business, threading his way through the streets without often stopping to recognize acquaintances. In common with the bulk of our population he shared to some extent in the disturbances of organic structures, during later December, and early January, from what was facetiously called "the Epizoty," but was not incapacitated for his daily routine of business, which he attended to personally, and without interruption, to the close of Wednesday, January 15th, 1873.

I noticed him on the bridge late Wednesday afternoon, and saw that his gait was unusually slow; and that his legs appeared to be too heavy for him to lift them easily in walking. He went to his

* A course of studies in the public schools of Prussia is designated "REALIEN," which may be translated "Realities," things as opposed to mere names.

residence rather earlier than usual that evening, undressed himself, and went to bed, saying he was very tired, and felt sore all over. He did not speak of having had a chill, and the family did not notice anything specially unusual. About 5 o'clock, P. M., I placed a thermometer in his exilla, and when it had settled it marked 107½°. I turned to Mrs. W. and said, "this means business." My attention was immediately turned to his chest, as it seemed to me he must have so called Pneumonia. I found some dullness low down on the right side, and the respiratory sounds were changed, indicating that a less volume of air than naturally was admitted in the act of breathing. In fact, that repair of structure, as it wastes in the performance of function, was partially, if not wholly arrested; and that existing structure was somewhat modified, as evidenced by modified function. The blood arriving there was unable to pass through this modified structure at physiological velocities; was detained, decreasing the area of the air tubes and vesicles, constituting the initial phenomena of so called pneumonia. The pulse did not correspond with the temperature, being only about 110, with moderate force and fulness, breathing 21 per minute.

The condition of things thus made known, was, to my mind, of a very grave character. Repair of structure, at the velocity of motion made known by the temperature, was simply impossible; and any remains of undigested food in stomach, or intestines, would undergo ordinary decomposition if retained, and its products add still further to the embarrassments of patients condition.

Therefore, he is to take a table spoonful Syr. Ipecac, with a tumbler water as hot as he can drink it after each dose; and the doses of Ipecac to be repeated every twenty minutes till he has vomited. When stomach has settled, to have four compound Cath. Pills, U. S. P. to move his bowels. To have, also, poultice of flax seed meal, one twelfth mustard, over right lung. No food to night. May have tumbler milk after bowels move.

I left my patient with a sense of oppression on my mind as to his condition.

Thursday morning, January 16th, 8¼ o'clock. Patient has had a restless night. Vomited, but less than Mrs. W. thought desirable. Has had one good motion from bowels, of a very offensive character. Temperature dropped down to 102°; pulse about 90, not accurately counted; breathing 22. Has some cough, but it is not troublesome. Complains of being very tired, and more or less stiff and sore all over.

His condition is manifestly better, but his pulse is very feeble, and is easily obliterated, though there is a good volume in the artery.

He is to take to day thirty drops Tinct. Digitalis, six grains Quinia S. every six hours. May have lemonade, solution chlorate potassa, (teaspoonful to pint water), as drinks; plain water sometimes if he desires it, food and milk punch.

5 O'clock P. M. Patient has not set up to day, only to have towel bath, his clothing changed and bed made. Has taken medicine and considerable bread and milk, but does not like sol. chlorate potassa. Does not feel any better of his weakness. Temperature 103, pulse 110, respirations 22.

To have to night, fifteen grains Dovers powder, and solution chloral hydrat, in ten grain doses in addition, sufficient to get a good nights sleep. Milk punch if he is awake.

Friday morning, January 17th, 8 o'clock. Mrs. W. says her husband did not sleep good, complained of pain over left eye about one o'clock in the morning. She had laid down beside him, and had the gas turned down, and did not turn it up but gave him another dose of chloral, after which he became more quiet and dozed off till 5 o'clock, when she turned the gas on, and discovered his left eye much swollen. It is now the size of half a hen's egg; cuticle gone on upper lid, and the surface apparently raw, with some matter oozing from the inner cauthus. Skin somewhat raised over temporal muscle, and slightly so on forehead. Vision was shut out from left eye. Right eye lid not much swollen, but perceptibly thicker. Complains that his head aches, and wants to know when it will get better. Temperature 103, pulse 115, resp. 22. Face as a whole perceptibly enlarged, as if seen through a magnifying glass.

Recognizing the gravity of my patients condition, I prescribed Glycerine ℥i. Crys. Carbolic Acid, ʒiss, to be applied to swollen parts with brush, and soft clothes, in small pieces, placed on them, and kept wet with the carbolic glycerole. Tinct. Ferri Mur. gtt. xv, and Quinia Sulph. gr. vj, every three hours. To have beef steak, beef essence, milk punch, and plain milk, all he can be induced to take, withont reference to regular meals. All this went into effect in half an hour.

2 O'clock P. M. Swelling apparently not extending; more matter oozing from inner corner of the eye. Temperature, pulse, and breathing, as in the morning. Has directed his business as usual to-day, had some business friends to call on him in addition. Continue treatment.

8 O'clock P. M. Has talked business all afternoon; made a will, and had it destroyed again; directs his business as in health. Would not allow cloths to remain on his face; but it was painted with the glycerole a number of times. Left eye lid more swollen. Face generally larger; more tumefaction about the forehead, though no fluctuation beneath integuments. Left ear quite large, and red; redness extends to neck on left side. Right side not red, and not much, if any, swollen. Right eye lid larger, though vision not shut out. Temperature 104, pulse 110, respiration 24. Pneumonic symptoms not prominent; no farther extention of dullness can be detected; and not much cough. Has taken largely of milk to-day; prefers it to anything else; had considerable beef essence, and has had medicine punctually. Complains that his head hurts him. Gas to burn all night, and he is not to be unobserved by wakeful eyes at any time during the night. As his bowels have not moved for 48 hours, and the temperature has been much above natural all that time, he is to have a wine glass full Sol. Saline Sulphates* in a tumbler water when he asks for a drink of water, at almost any time during the night. Continue other treatment as during the day, if he is awake. Not to be waked for anything if if he sleeps. Chloral and Dovers powder, as last night, to procure sleep.

Saturday morning January 18th, 8 o'clock. Patient did not sleep much last night; dozed many short naps. Had a motion from bowels late in the night. Right eye closed by enlargement of lid;

* Buffalo Medical and Surgical Journal, May 1872, p. 3[illegible]0.

he is therefore blind. Was asleep much of the time between six and eight o'clock this morning. When he ascertained the time, he demanded to have the foremen of his business brought to him, to give them instructions for the day; and would not do anything else till they came, and he had told them what to do. Temperature 103; pulse and respiration as last evening. Left eye lid and ear covered with diptheritic exudation. Intellect clear; hearing good. Except the extension of the swelling in the right eye lids, he seems to be holding his own. He is to have food more systematically than yesterday; same medicine punctually as yesterday. To be asked to pass water every six hours, if he does not do so of his own accord.

2 O'clock P. M. Much as at morning visit. Business friends have been with him all morning, talking business, and receiving instructions what to do in case he should not recover. Continue treatment.

8 O'clock P. M. Temperature 104, pulse and respirations as at last visit. Business friends have been with him all afternoon, one of them requested Mrs. W. to mention to me that cranberry poultices were a sure cure for erysipelas. Wanted them applied, whether or no, in my absence; but they were not put on. There has been some extension of the puffiness under scalp; and there is matter oozing from the left ear; otherwise he seems to be holding his own.

To have the gas burn, and watchers all night; to have Dovers powder and Chloral Hydrat to procure rest and sleep. Food and medicine if awake, as during the day.

Sabbath morning, January 19th, 8 o'clock. Patient has passed another restless night, has passively delirious part of the time. Face and scalp enormously swollen this morning. The entire scalp seems lifted from its attachments to the cranium; and the crackling sound on pressure announces the pressure of gas beneath—Emphysema, so called. Neck, as well as face, also, greatly enlarged. Temperature 103, pulse 120, respirations 36 per minute, rapidly running up to 60, two hours later.

A consultation was held at 9 o'clock A. M. I proposed some incisions in the scalp with a view of giving exit to the effusion beneath it; and one was made just below the eye brow of the left eye, but little or nothing escaped, and no other made. The crackling beneath the scalp, told but too plainly that ordinary putrefac_ tive decomposition was at work, and its products, liquid and gaseous, were confined beneath the integument. In the consultation no other treatment was recommended than that he was getting, but the application of cranberry poultices to the face and scalp, more to satisfy friends, than any hope or expectation of benefit. The cranberries were softened by heat, and applied at 10 o'clock, in gauze bags. In a quarter of an hour after their application all circulation in scalp and face ceased. The poultices were removed, and hot applications made to the face and scalp, with the purpose of re-establishing the circulation. At the end of two hours they were discontinued, without the end having been accomplished. The patient begged to be let alone, and beyond giving him water, nothing more was attempted, and he expired about 3 o'clock P. M.

The profession calls this a case of Erysipelas, phlegmonous erysipelas; and it will appear as such in our Slender Mortuary records at the cemetery. And the people, taught by the profession, will also call it erysipelas. And in so far as the name leads to conceptions of a something not natural to the body, or foreign to it, having brought about the modifications of structured forms to which this particular name or term is applied, it is misleading. Nor were the pathological conditions limited to the face, scalp, and neck, as evidenced by the temperature of the body in the axilla.

In looking over a translation of the curriculum of study in the public school of the new German Empire, recently, I found one course of studies named "REALIEN." The translator, in a foot note, says, there is no english word which exactly reproduces the original; but that it means realities of things disconnected from the names, or terms by which they are known. Without any details as to what is taught as Realien in the Prussian public schools, I cannot give its scope, but I nevertheless think the purpose of teaching the realities of things, separate from their names, a valuable advance in the course of studies in public schools in any country.

It seems to me that medicine sorely needs the *Realien.* The world has outgrown its ponderous technicalities, and minute classifications, which ought now to be abandoned, or have interpreters of their Realien. No other department of human knowledge is now so encumbered. The schisms in its ranks, and the numerous sects into which it is divided, who go before their fellow men with the acknowledged catalogue of "diseases" in one hand; and the still more numerous catalogue of "drugs and medicines" for their cure in the other, asking their fellow men to test their skill, is a humiliating spectacle indeed. The honor and dignity of the regular profession, as well as the good of their fellow men, alike concur in showing the necessity for a speedy reconstruction of its elementary branches on REALIEN.

The case just reported I consider to have been one of more than ordinary interest, studied, as it should be, on its facts, its *realien,* and not upon its name, or nosology.

After the demise of Mr. W., Mrs. W. recalled to her memory that a number of days before he came to his bed he asked her several times why his left eye and forehead were so tender? and why little pains would dart through them? and further, why his face smarted when he came to the fire? In reply, she thinks she told him it was owing to the cold weather, and that it would soon be well, and so dismissed the matter from her mind. In scanning him critically after he came to his bed I certainly did not notice any special changes in the integuments which so suddenly gave way on Friday morning, January 17th. But they did not give way without adequate cause, and it is for myself, as a student of the phenomena of life, their REALIEN, to obtain a satisfactory solution to the problem offered by his case.

I may remark that the cause, or causes which give rise to, and maintain life, those that disturb it; and those that terminate it, are to be looked for just where life is found; among the forces, materials, and conditions surrounding living beings.

The condition of the weather, that is, the earths forces, during the preceeding month, and in fact since the close of last autumn, has been exceptionally peculiar in most respects. The mean temperature has been lower than for many years past; and the fluctuations of temperature have been sudden and violent in the extreme, sometimes amounting to 50° Fah. in 24 hours. About the first of January several members of Mrs. W's family were taken sick. They called it the Epizootic; but the facts were that the exposed structures of the mouth, nose, and lungs, were so modified that their rate of waste was greater than their rate of repair, as evidenced by modified functions; and that these modifications were not confined to them, the elevation of the temperature of the whole body, was conclusive evidence. His eldest daughter, after a chill, had, what they called "Nueralgia of the face," and was confined to bed an entire week; the temperature reaching 104° at one time. One after another the remaining members of the family were, in many respects, similarly sick, and none of them had wholly recovered when Mr. W. died.

Then, coming to Mr. W. his habits and mode of life throw much light on the phenomena of his case. A horse, kept for his personal service, died early in December with the Epizootic. And as all the horses in the city and adjoining country were sick, he could not buy one, or hire one, and so, had to walk all the time, and in all conditions of weather, to transact his large and scattered business. Thus, every monday morning he left home at peep of day, and aimed to visit every grocer in the city with whom he had dealings, settle for last week's business, and be at home at or before 11 o'clock in the morning. He had so much business, and attended to so much of it personally, that he was much exposed. And he was by no means the most careful man about his own protection against cold and wet. He had, it is true, everything to live with, or could get it, bnt did not think any extra care of his own person necessary; and, therefore, seldom took any. He required his family to use their wraps and over shoes, but did not attend to himself.

It is, to my mind, conclusively shown by the events preceding, and succeeding, his brief, and last illness, that his face had been partially frozen a number of times, but not sufficiently so unhappily as to demand immediate attention for, in that case, I think it among the possibilities, that he would now be among us living; but sufficiently so to modify, to some extent, the molecular arrangement of the material of his structure about the exposed parts of his face, not sufficient at any one time to suspend function, but to impair it. Repair in normal molecular arrangement was certainly retarded and modified, if not arrested, And their reproduction in normal molecular arrangement was not fully provided for in their acts of functional decay.* The excessively high temperature of the whole body disclosed by the thermometer, Wednesday evening, January 15th, announced some coming, and grave mischief, in his structures An examination of his lungs makes known the arrest of repair there, and resulting modification of structure.

Thursday morning, in consequence, to some extent, perhaps of the better conditions of life surrounding him—in doors, and bed—his downward progress is, apparently, retarded, even seeming better, though his soft pulse indicated decreasing power to carry on the circulation; and to increase which, by advancing the rate of repair in structure, the Digitatis, Quinia and milk punch, &c., were given. But the story was very brief, for Friday morning discloses the fact, though not at that moment required, that putrefactive decomposition had commenced in his eye lids, and beneath adjoining integuments. Nor was his progress downward rapid on Friday—apparently stayed, again, possibly, by the remedial measures employed, and an extension of ordinary decay is visible in the swelling of left ear, right eye lids, and lips, Saturday morning. During Saturday progress again apparently stayed; but Sabbath morning reveals his moribund condition, and death in the afternoon.

Does the term Erysipelas, or phlegmonous Erysipelas, or even moist gangrene, convey any proper conception of the *Realien* of his case ? I think not.

On the outside, here were occurring just such pathological changes in structure, as take place in occluded cavities; as the cranium chest and abdomen, as demonstrated by autopsies within reach of inspection and study by unassisted vision. What were they ? Not irritation; not inflammation;—the fiery god of pathology—not erysipelas; not phlegmonous erysipelas; not moist gangrene, but there was a speedy—rapid—return of the materials of structure from their complex condition in living flesh, to their ordinary states—simpler chemical conditions—in inorganic nature,

* See Buffalo Medical and Surgical Journal, July 1871, p. 449.

by what is known as putrefactive decomposition, just such as would have occurred two or three days after death by violence in a July temperature of 90°.

True, there was what is called irritation, inflammation, diphtheritic exudation, erysipelas, phlegmonous erysipelas, moist gangrene, or sphacelus, as they are called, all open to study, by the naked eye. But they are not *Realien.* What was transpiring, and had already transpired, in Mr. W's structures, more prominently about the face, scalp, and neck, was the rapid descent of matter from its chemical complexity in living structures, through various chemical mutations, to its ordinary, and simpler chemical conditions, in inorganic nature. That it seems to me is the *Realien.*

The apparently living structures perform no function, and do not in the act of decay provide for their own reproduction in natural structural arrangement from new material. The more unstable structures under the denser integuments give way first, and the liquid and gaseous products of decay are confined beneath them, giving rise to the what is called Emphysema; and to give exit to which incisions were proposed, one made, and as nothing escaped, gave warning, which was heeded, to make no more.

Did the treatment instituted in Mr. W's case do any good? Possibly it may have delayed the close of life, but even this is doubtful. It was, it is true, such as experience has shown to be followed by the best attainable results; but in the present case, any good results, are, at least, involved in doubt. And with the light which retrospection adds to our studies of such phenomena, I do not now see how it could have been bettered. True, remedial agents were prescribed, not with a view of *curing* my case, but to retard motion of matter, and direct force towards the reproduction, and repair of wasting structures, from new material; and to give exit to effete matter from his body. It has afforded me a very interesting study, such as was never before presented, since I began to look at, and study things, outside of names—THEIR REALIEN.

